# Diagnostic accuracy and repeatability of ChatGPT using textual and radiographic data in reversible pulpitis: a retrospective diagnostic study

**DOI:** 10.3389/fbinf.2026.1877345

**Published:** 2026-07-21

**Authors:** María Llorente de Pedro, Yolanda Freire, Natalia Moneo, Cristina Andreu-Vázquez, Roberto Estévez, Víctor Díaz-Flores García, Ana Suárez

**Affiliations:** 1 Department of Dentistry, Faculty of Biomedical and Health Sciences, Universidad Europea de Madrid, Villaviciosa de Odón, Spain; 2 Department of Veterinary Medicine, Faculty of Biomedical and Health Sciences, Universidad Europea de Madrid, Villaviciosa de Odón, Spain; 3 Universidad Europea de Madrid, Faculty of Biomedical and Health Sciences, University Dental Clinics, Villaviciosa de Odón, Spain

**Keywords:** ChatGPT, conservative dentistry, dentistry, diagnostics, LLM, reversible pulpitis

## Abstract

**Objectives:**

Large language models have recently evolved into multimodal systems capable of interpreting images alongside text. The aim of this study was to assess the diagnostic accuracy and repeatability of ChatGPT-5 for reversible pulpitis, based on structured clinical information and periapical radiographs (PRs).

**Methods:**

Thirty expert-validated cases of reversible pulpitis were retrospectively selected. For each case, clinical data and PRs were compiled to create a reference dataset, with expert consensus serving as the reference standard. A structured prompt incorporating clinical information and PR was designed and included six predefined questions: four addressing diagnostic reasoning, one assessing whether PR information was used to establish the diagnosis, and one requiring radiographic findings description. Each case was entered thirty times into ChatGPT-5, generating a total of 900 answers. Answers were assessed by two independent experts, with disagreements resolved by a third. Radiographic descriptions were graded using a three-point Likert scale. Diagnostic accuracy and repeatability were analysed using binomial and agreement methods.

**Results:**

Diagnostic questions related to tooth identification, pulpal diagnosis, periapical diagnosis and treatment indication showed accuracies above 96%. Overall accuracy for radiographic description was low (4.67%). Coronal radiographic interpretation showed poor performance (5.15%), whereas accuracy for pulpal and periapical structures exceeded 96%. Repeatability was almost perfect for diagnostic questions and pulpal/periapical interpretation, but moderate for coronal descriptions.

**Discussion:**

ChatGPT-5 demonstrated high accuracy and repeatability in text-based diagnostic reasoning for reversible pulpitis. However, limitations in radiographic interpretation, particularly of coronal structures, indicate that its use should be limited to a supervised decision-support role.

## 1 Introduction

An accurate clinical diagnosis requires understanding the relationship between biological conditions and clinical and radiographic findings (Azim et al., 2022). In caries management, radiographs are used for diagnosis (Mohamad et al., 2022) and assist in estimating the depth of demineralization within enamel and dentin (Ismail et al., 2015). However, as the caries process progresses, pulpal involvement may occur, making the determination of pulp status necessary for establishing an appropriate treatment plan (Zero et al., 2011). In such cases, radiology also plays a fundamental role (Patel et al., 2019; Setzer and Lee, 2021) in diagnosis, treatment planning, intervention execution, and follow-up assessment of therapeutic outcomes (Lo Giudice et al., 2018). Among the different radiographic techniques available in dental practice, periapical radiography (PR) remains an important diagnostic tool across multiple clinical contexts (Cotti and Schirru, 2022). It is often the initial imaging modality for evaluating periapical lesions (Patel et al., 2019) and is also used to assist in the diagnosis of caries (Lin et al., 2022).

The integration of radiographic imaging with artificial intelligence (AI) systems has recently begun to transform the clinical environment (Najjar, 2023), providing alternative approaches to diagnostic analysis and report generation (Balel et al., 2025; Stephan et al., 2024). Convolutional neural networks (CNNs) demonstrate strong performance in extracting features from medical images (Azad et al., 2022) and are among the most widely used algorithms for image recognition and analysis tasks (Ker et al., 2018). They have enabled the development of AI-driven diagnostic platforms, such as Diagnocat (Diagnocat Inc., San Francisco, CA, United States of America) (Allihaibi et al., 2025). Studies evaluating the performance of such platforms on PRs suggest they may support the diagnosis of dental caries (Szabó et al., 2024) and periapical radiolucent lesions (Allihaibi et al., 2025; Issa et al., 2023), even though these systems are specifically trained for image-based pattern recognition tasks.

However, although CNNs have long dominated image analysis (Azad et al., 2022; Nirthika et al., 2022) large language models (LLMs), which were initially designed for text-based tasks, have recently evolved into multimodal systems capable of interpreting text, images and other types of data (Durmazpinar and Ekmekci, 2025; Meskó, 2023). Contrary to CNNs, multimodal LLMs combine pretrained vision models and language models, handling interleaved image/text sequences (Alayrac et al., 2022). Recent models, like ChatGPT-5, are trained to think before they answer: they can produce a long internal chain of thought before responding to the user. The models learn to refine their thinking process, try different strategies, and recognize their mistakes. Moreover, these models further extend this capability by thinking with images in their chain-of-thought (Singh et al., 2026). Therefore, advanced LLMs like ChatGPT-5 offer an integrated framework that merges visual and linguistic analytics capabilities, thus streamlining the interpretative process of medical images (Hu et al., 2024).

Their increasing accessibility and broad applicability highlight the importance of investigating their performance in these emerging tasks, particularly since their core architecture is not primarily optimized for visual feature extraction and most current literature only examines text-based capabilities. One of the most widely used LLMs, ChatGPT (OpenAI, San Francisco, CA), has been evaluated in areas such as endodontics (Abdulrab et al., 2025; Kark et al., 2024) oral surgery (Balel et al., 2025; Alsayed et al., 2024) periodontology (Alan and Alan, 2023) or preventive dentistry and Oral Oncology (Alsayed et al., 2024). Reported performance varies, likely due to differences in clinical domains and methodological design.

By contrast, few studies have evaluated the performance of LLMs in image-based diagnostic interpretation. In medicine, evaluations have included the assessment of radiograph quality (Arruzza et al., 2024), BI-RADS ultrasound interpretation (Liu et al., 2025), the interpretation of abdominal computed tomography and magnetic resonance imaging scans (Elek et al., 2024), and neurosurgery board examination images (Sau et al., 2025). In Dentistry, studies have assessed ChatGPT’s interpretation of panoramic radiographs (Silva et al., 2024; Suárez et al., 2025) and its diagnostic accuracy in endodontic scenarios (Durmazpinar and Ekmekci, 2025). As with text-based tasks, the findings have varied among the studies.

Consequently, it is important to evaluate the performance of LLMs such as ChatGPT in emerging diagnostic capabilities, including radiographic analysis (Durmazpinar and Ekmekci, 2025; Meskó, 2023). Given its potential use as a support tool for clinicians when interpreting textual and radiographic information, it is particularly relevant to assess accuracy and repeatability of ChatGPT’s performance in clinical reasoning and image interpretation. In this context, ChatGPT may be considered by some clinicians as a decision-support tool, highlighting the need to evaluate its diagnostic performance, particularly in challenging diagnostic areas such as pulpal diseases (Raoof et al., 2022). In cases of reversible pulpitis, distinguishing it from more advanced pulpal conditions may determine whether a conservative approach or endodontic treatment is indicated (Donnermeyer et al., 2023; Sabeti et al., 2026). Therefore, the emergence of LLMs offers new opportunities to support these diagnostic processes through the simultaneous integration of clinical and radiographic information, making them a potentially valuable auxiliary tool for clinical decision support.

Despite the growing interest in multimodal large language models LLMs, evidence regarding their diagnostic performance using combined clinical information and periapical radiographs remains limited. Because image analysis capabilities have only recently been incorporated into LLMs, most previous studies have focused on text-based tasks. In addition, evaluating the repeatability of LLM-generated answers is essential for accurately assessing model performance. Consequently, the reliability and potential clinical role of these models as decision-support tools remain uncertain. To address these challenges, this study assessed the diagnostic accuracy and repeatability of ChatGPT-5 using expert-validated cases of reversible pulpitis that included structured clinical information and periapical radiographs.

Thus, the aim of this study was therefore to evaluate the accuracy and repeatability of ChatGPT-5’s diagnostic reasoning related to reversible pulpitis, based on textual information and periapical radiographs, using expert assessment as the reference standard. The null hypothesis was that there would be no differences in diagnostic reasoning related to reversible pulpitis between ChatGPT-5 and clinical experts.

## 2 Materials and methods

### 2.1 Ethical approval

This study was conducted in accordance with the ethical principles of the Helsinki Declaration and current legislation on research involving human participants. Ethical approval was granted by the Ethics Committee for Research with Medicines (CEIm) of Hospital Universitario de Getafe (Approval Code: CEIm25/117). Data collection complied with Regulation (EU) 2016/679 on the protection and free movement of personal data. Written informed consent was obtained from all participants, authorizing the inclusion of anonymized data extracted from their medical records for research purposes in the study.

### 2.2 Clinical cases selection

**FIGURE 1:**
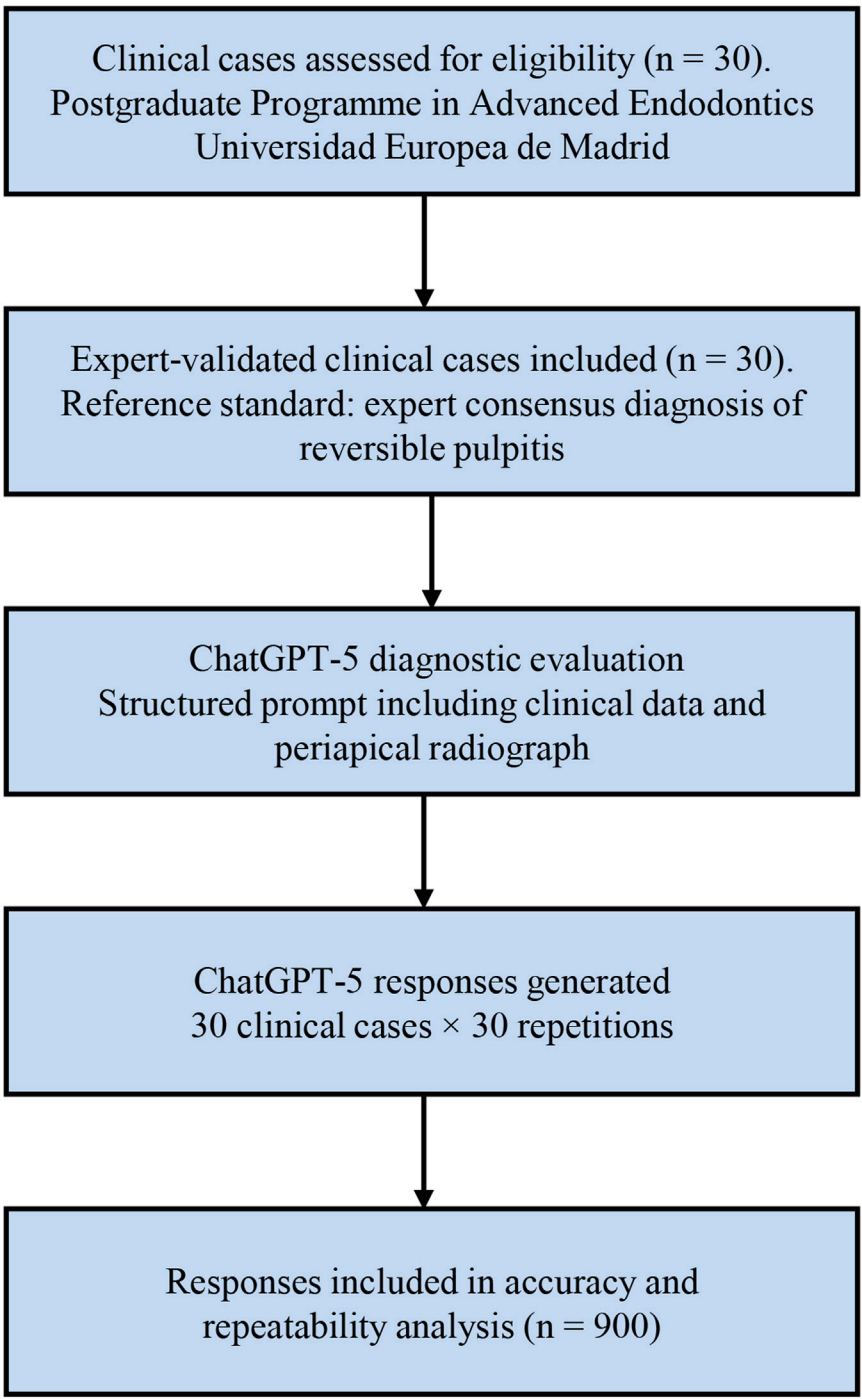
STARD flow diagram of clinical case selection and analysis. The unit of analysis was expert-validated clinical cases and model-generated responses. No patient recruitment, follow-up, or clinical interventions were involved.

This study followed a retrospective diagnostic accuracy design. In June 2025, two experienced endodontic experts (V.DF.G. and R.E.) selected a sample of 30 clinical cases from a Postgraduate Programme in Advanced Endodontic Programme of the University Master’s Degree in Advanced Endodontics (Universidad Europea de Madrid). Cases were identified based on a prior expert-established diagnosis of reversible pulpitis. The endodontic diagnosis was established according to the recommended diagnostic terminology of the AAE Consensus Conference Recommended Diagnostic Terminology (AAE, 2009), whereas caries-related findings were classified according to the consensus documents on deep caries (Bjørndal et al., 1999; Innes et al., 2016). A convenience sample of expert-validated clinical cases was used. The flow of clinical case selection and analysis is shown in Figure 1.

Eligibility cases included patients aged over 18 years, availability of periapical radiographs of adequate quality, and the presence of a single tooth with suspected endodontic pathology. Periapical radiographs were acquired using the CS 2100 system (Carestream Health, Inc., New York, United States of America) at 60 kV and 7 mA. The PRs were captured using storage phosphor plates (SPPs) and subsequently processed using the VistaScan Mini system (Dürr Dental Medics Iberica S.A.U., Barberà del Vallès, Spain). All radiographs used in this study were original and had not undergone any additional digital processing beyond the manufacturer’s standard acquisition workflow. All clinical and radiographic data were anonymized prior to analysis and publication by removing any potentially identifiable patient information.

The sample size was defined in advance based on previous studies that evaluated the accuracy and repeatability of large language models in dental and medical contexts (Suárez et al., 2025). In line with these studies, a limited number of expert-validated clinical cases were selected as the focus of the analysis was the model-generated answer rather than the individual patient. Assuming expected accuracy and repeatability values of approximately 90%, a 95% confidence level, and a margin of error of ±2%, the minimum sample size was 864 observations. Including 30 clinical cases, each evaluated through 30 iterations (yielding 900 outputs), met this requirement and enabled a robust assessment of the model’s answer variability, repeatability and internal consistency, while ensuring comparability with previous studies in this field.

### 2.3 Database preparation

Data from the 30 cases were systematically recorded in a Microsoft Excel (Microsoft Corp., Redmond, WA, United States of America, RRID:SCR_016137). The following parameters were documented for each case: (i) the tooth with suspected pathology, (ii) pulp sensitivity response, (iii) percussion test results, (iv) the indication of root canal treatment, and (v-vi) radiographic findings that support the diagnosis. This expert-validated dataset was used to construct the prompt and served as the reference standard (ground truth) to evaluate ChatGPT’s answers. Expert consensus was chosen as the reference standard because no objective gold standard exists for the diagnosis of reversible pulpitis, and expert assessment represents the accepted clinical standard.

### 2.4 Prompt design

A structured prompt was designed based on the reference dataset. The model role was first defined, followed by specification of the input types (clinical data and PR image). Next, the pulp sensitivity and percussion findings for each case were incorporated. To standardize the output format across repetitions, the model was instructed to respond directly to six predefined diagnostic questions using concise answers without explanatory text. The corresponding PR was included in the same input. The same standardized prompt was used across all iterations. ChatGPT-5 had access only to the clinical information and periapical radiographs provided in the prompt and had no access to the expert reference diagnoses.

**FIGURE 2:**
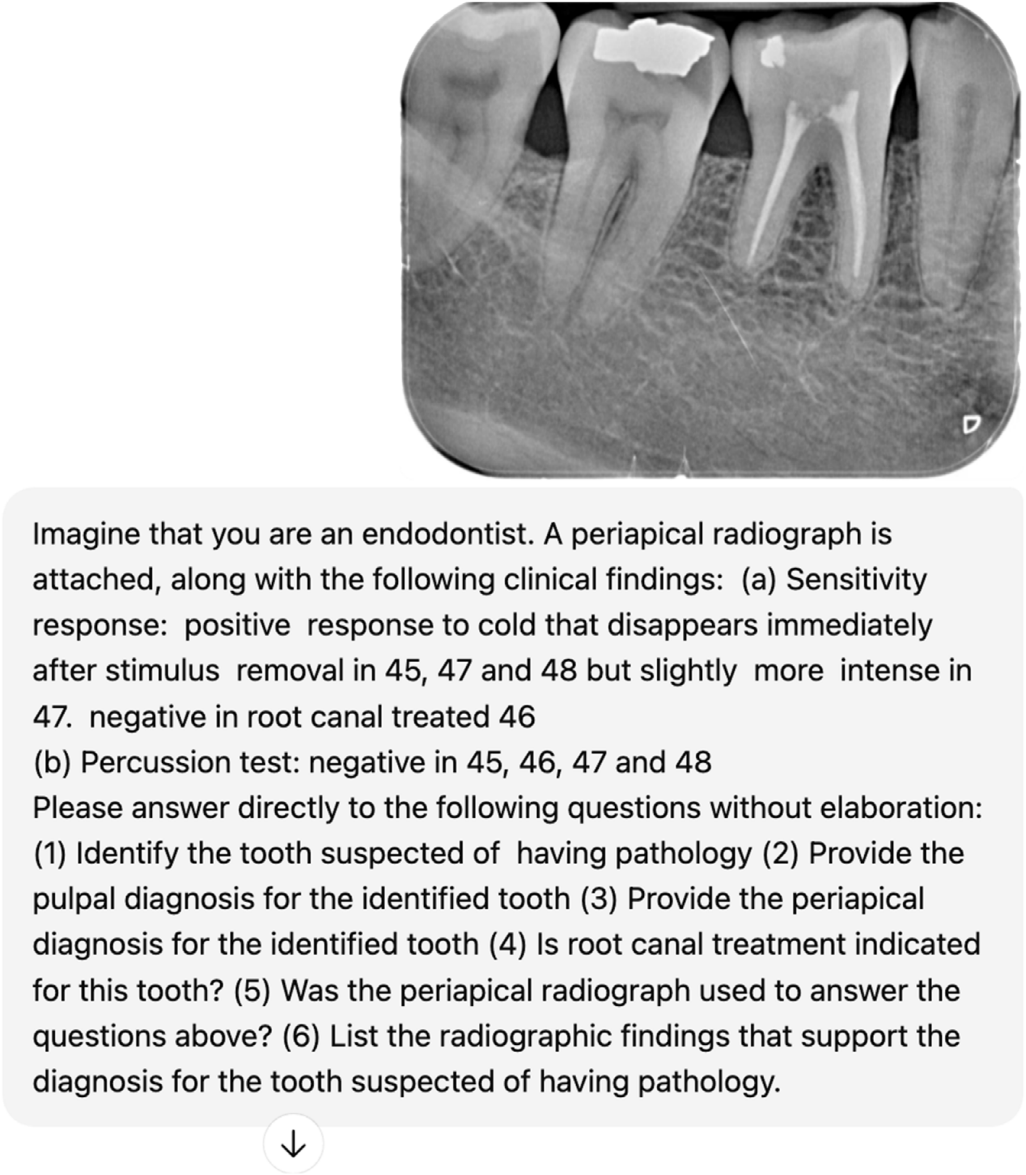
Screenshot of the prompt entered into ChatGPT for Case 27.

The six predefined questions were the following: Question 1 (Q1): Identify the tooth suspected of pathology; Question 2 (Q2): Provide the pulpal diagnosis for the identified tooth; Question 3 (Q3): Provide the periapical diagnosis for the identified tooth; Question 4 (Q4) Is root canal treatment indicated for this tooth?; Question 5 (Q5): Was the periapical radiograph used to answer the questions above?; Question 6 (Q6): List the radiographic findings that support the diagnosis for the tooth suspected of pathology. The prompt used in one of the clinical cases can be seen in Figure 2.

### 2.5 Answer generation

The answer generation procedure was adapted from a previous study (Suárez et al., 2025) and modified to align with the specific objectives of this study. Two independent ChatGPT Plus accounts were used. Each clinical case was entered 30 times via the ‘new chat’ function in temporary sessions (Kung et al., 2023; Shieh et al., 2024), and the chat history was deleted after each iteration. This minimized the effects of conversational memory and contextual carryover between iterations.

To minimize potential temporal variability in model behavior, answer generation was distributed across three time slots (morning, afternoon, and evening). Answers were produced between 1 and 15 October 2025 yielding 900 answers (30 cases × 30 iterations) with ChatGPT-5 (Thinking mode, OpenAI, San Francisco, CA, United States of America, RRID:SCR_023775). All answers were transferred to the reference dataset spreadsheet (Excel).

### 2.6 Answer assessment

ChatGPT-5 answers were classified into predefined categories based on concordance with the expert reference standard. Answer assessment was performed by experts different from those involved in case selection and ChatGPT-5 answer generation. Two endodontic experts (M.Ll.P and N.M.) independently assessed the 900 sets of answers against the reference dataset. Questions 1 to 4 (Q1-Q4) were scored dichotomously (0 = incorrect; 1 = correct). Answers were considered correct when they fully matched the expert diagnosis and incorrect otherwise. Question 5 (Q5) was not evaluated, as it served a descriptive purpose. Question 6 (Q6) was initially scored dichotomously (0 = incorrect; 1 = correct). Subsequently, the description of the coronal part (Q6A) and the periapical/pulp part (Q6B) were evaluated independently using a three-point Likert scale (Table 1). In cases of disagreement, a third independent expert (Y.F.), different from the initial raters, resolved the discrepancies. Reference standard classifications were based on predefined expert diagnostic criteria, with expert consensus considered definitive and no alternative categorization applied.

**TABLE 1:** Expert criteria for assessing ChatGPT answers to the Q6A and Q6B components of Question 6.

Assesment	Description
Incorrect	The answer generated is incorrect or unrelated to the question. It does not demonstrate an adequate understanding or knowledge of the topic.
Correct but partially incomplete	The answer generated shows some understanding or knowledge of the topic, but there are missing elements. Although the information is correct, the answer is not complete enough to be considered true or adequate
Correct	The answer is completely accurate and shows a solid and precise understanding of the subject. All components are addressed in a thorough and accurate manner

### 2.7 Statistical analysis

ChatGPT-5 performance was assessed in terms of accuracy and repeatability. Relative frequencies (n) and percentages (%) were calculated for each assessment category (correct, incorrect, and correct but partially incomplete for Q6A and Q6B). Analyses of diagnostic accuracy were pre-specified and performed separately for each predefined question and, for Question 6, for the coronal and pulpal/periapical components. No indeterminate results were recorded for either the index test or the reference standard. No missing data were observed for either the index test or the reference standard.

Accuracy was calculated overall and for each question individually and was defined as the proportion of answers graded as correct, with 95% confidence intervals estimated using the Wald binomial method. Repeatability was evaluated using percentage agreement and Gwet’s AC coefficient. Interpretation followed Gwet’s scale (Gwet, 2014): <0.0 Poor, 0.0–0.2 Slight, 0.2–0.4 Fair, 0.4–0.6 Moderate, 0.6–0.8 Substantial, 0.8–1.0 Almost Perfect. Analyses were performed using STATA software (STATA version BE 14; StataCorp, RRID:SCR_012763).

## 3 Results

In total, 900 sets of answers (thirty cases, thirty repetitions with six questions) were generated using ChatGPT-5 and evaluated. The overall accuracy of ChatGPT across the different answers is shown in Table 2. Questions Q1, Q2, Q3, Q4, and Q6B showed an accuracy above 96%. In contrast, questions Q6 and Q6A showed an accuracy below 5%. ChatGPT reported having used the provided radiograph to answer the questions in 95.56% of cases, with a 95% confidence interval of 94.11%–96.9%.

**TABLE 2:** Overall accuracy of ChatGPT answers for each question.

Question	Accuracy	IC 95% (Wald binomial)	
Q1	97.11%	96.02%	98.21%
Q2	96.56%	95.36%	97.75%
Q3	97.11%	96.02%	98.21%
Q4	97%	95.89%	98.11%
Q5	95.56%	94.21%	96.90%
Q6	4.67%	3.29%	6.04%
Q6A	3.89%	2.63%	5.15%
Q6B	96.56%	95.36%	97.75%

The repeatability of the answers varied according to the question and is presented in Table 3. For questions related to the diagnosis, repeatability was classified as almost perfect. Regarding the grading of the experts’ radiographic descriptions, repeatability was almost perfect for the pulpal/periapical component, whereas it was moderate for the description of the coronal component.

**TABLE 3:** Expert agreement for each question.

Question	Percent agreement (95% CI)	Benchmark	Gwet’s AC (95% CI)	Benchmark
Q1	98.1 (95.5–100.0)	Almost perfect	98.0 (95.0–100.0)	Almost perfect
Q2	97.0 (94.2–99.9)	Almost perfect	96.8 (93.5–100.0)	Almost perfect
Q3	98.1 (95.5–100.0)	Almost perfect	98.0 (95.0–100.0)	Almost perfect
Q4	97.9 (95.3–100.0)	Almost perfect	97.8 (94.8–100.0)	Almost perfect
Q5	95.1 (92.0–98.2)	Almost perfect	94.7 (90.8–98.5)	Almost perfect
Q6	95.1 (91.9–98.2)	Almost perfect	94.6 (90.7–98.5)	Almost perfect
Q6A	77.1 (70.2–84.1)	Substantial	62.1 (46.2–78.0)	Moderate
Q6B	99.0 (98.1–99.9)	Almost perfect	98.93 (97.87–100.0)	Almost perferct

Benchmark scale: Poor <0.0, Slight 0.0–0.2, Fair 0.2–0.4, Moderate 0.4–0.6, Substantial 0.6–0.8, and Almost Perfect 0.8–1.0. CI, confidence interval. The distribution of expert grading is shown in Table 4 for questions Q1 to Q6, and in Table 5 for the analysis of question Q6, Q6A and Q6B. The percentage of correct answers ranged from 0% to 100%, depending on the case and the question. For Q1, Q2, Q3, and Q4, answers graded as correct showed 100% repeatability in 26, 23, 23, and 25 of the cases, respectively. For these questions, there were no cases graded as incorrect with 100% repeatability. For Q5, in 11 cases ChatGPT indicated that the PR was used to establish the diagnosis in all 30 repetitions. There were no cases in which the PR was reported as not being used for diagnostic purposes. For Q6, answers graded as incorrect showed 100% repeatability in 18 of the cases, with no instances of 100% repeatability for correct answers. For the subanalysis of Q6, Q6A showed 100% repeatability of incorrect answers in 7 of the cases, with no instances of 100% repeatability for answers graded as correct or partially correct. Q6B exhibited 100% repeatability of correct answers in 22 cases, with no instances of 100% repeatability for answers graded as incorrect or partially correct.

**TABLE 4:** Distribution of expert assessment for ChatGPT generated answers to Questions 1 to 6.

	Q1		Q2		Q3		Q4		Q5		Q6	
Case	n Correct	% Correct	n Correct	% Correct	n Correct	% Correct	n Correct	% Correct	n Yes	% Yes	n Correct	% Correct
Case 1	30	100	30	100	30	100	30	100	29	96.67	1	3.33
Case 2	30	100	29	96.67	30	100	30	100	30	100	0	0.00
Case 3	29	96.67	29	96.67	29	96.67	29	96.67	29	96.67	0	0.00
Case 4	30	100	30	100	30	100	30	100	30	100	4	13.33
Case 5	30	100	29	96.67	30	100	29	96.67	30	100	0	0.00
Case 6	7	23.33	7	23.33	7	23.33	7	23.33	7	23.33	0	0.00
Case 7	29	96.67	29	96.67	29	96.67	29	96.67	29	96.67	2	6.67
Case 8	30	100	30	100.00	30	100	30	100	30	100	0	0.00
Case 9	30	100	30	100.00	30	100	30	100	30	100	0	0.00
Case 10	29	96.67	29	96.67	29	96.67	29	96.67	29	96.67	4	13.33
Case 11	30	100	27	90.00	30	100	30	100	30	100	1	3.33
Case 12	30	100	30	100	30	100	30	100	30	100	0	0.00
Case 13	30	100	30	100	30	100	30	100	30	100	0	0.00
Case 14	30	100	30	100	30	100	30	100	30	100	1	3.33
Case 15	30	100	30	100	30	100	30	100	26	86.67	0	0.00
Case 16	30	100	30	100	30	100	30	100	29	96.67	0	0.00
Case 17	30	100	30	100	30	100	30	100	29	96.67	1	3.33
Case 18	30	100	30	100	30	100	30	100	30	100	24	80
Case 19	30	100	30	100	30	100	30	100	30	100	0	0.00
Case 20	30	100	30	100	30	100	30	100	29	96.67	1	3.33
Case 21	30	100	30	100	30	100	30	100	30	100	1	3.33
Case 22	30	100	30	100	30	100	30	100	28	93.33	0	0.00
Case 23	30	100	30	100	30	100	30	100	30	100	0	0.00
Case 24	30	100	30	100	30	100	30	100	29	96.67	1	3.33
Case 25	30	100	30	100	30	100	30	100	29	96.67	0	0.00
Case 26	30	100	30	100	30	100	30	100	28	93.33	0	0.00
Case 27	30	100	30	100	30	100	30	100	30	100	0	0.00
Case 28	30	100	30	100	30	100	30	100	30	100	1	3.33
Case 29	30	100	30	100	30	100	30	100	30	100	0	0.00
Case 30	30	100	30	100	30	100	30	100	30	100	0	0.00

**TABLE 5:** Distribution of expert assigned grades for ChatGPT generated responses to the coronal (Q6A) and pulpal/periapical (Q6B) components of Question 6.

Question	Q6A				Q6B			
Case	n	%	n	%	n	%	n	%
	Correct	Correct	Partially correct	Partially correct	Correct	Correct	Partially correct	Partially correct
Case 1	0	0	17	56.67	30	100	0	0
Case 2	0	0	0	0	30	100	0	0
Case 3	0	0	2	6.67	29	96.67	0	0
Case 4	1	3.33	11	36.67	30	100	0	0
Case 5	0	0	8	26.67	29	96.67	0	0
Case 6	0	0	3	10	7	23.33	0	0
Case 7	2	6.67	18	60	29	96.67	0	0
Case 8	0	0	29	96.67	30	100	0	0
Case 9	0	0	0	0	30	100	0	0
Case 10	4	13.33	2	6,67	29	96.67	0	0
Case 11	1	3.33	6	20	30	100	0	0
Case 12	0	0	4	13.33	30	100	0	0
Case 13	0	0	9	30	30	100	0	0
Case 14	1	3.33	1	3.33	28	93.33	0	0
Case 15	0	0	6	20	30	100	0	0
Case 16	0	0	0	0	30	100	0	0
Case 17	1	3.33	12	40	30	100	0	0
Case 18	24	80	0	0	30	100	0	0
Case 19	0	0	3	10	30	100	0	0
Case 20	0	0	2	6.67	30	100	0	0
Case 21	0	0	12	40	29	96.67	0	0
Case 22	0	0	0	0	30	100	0	0
Case 23	0	0	0	0	30	100	0	0
Case 24	1	3.33	4	13.33	30	100	0	0
Case 25	0	0	18	60	29	96.67	1	3.33
Case 26	0	0	2	6.67	30	100	0	0
Case 27	0	0	25	83.33	30	100	0	0
Case 28	0	0	21	70	30	100	0	0
Case 29	0	0	0	0	30	100	0	0
Case 30	0	0	0	0	30	100	0	0

Correct: correct and complete; Partially correct: Correct but partially incomplete. Q6A: Question 6A, coronal answer evaluation for Question 6; Q6B: Question 6B, pulpal/periapical answer evaluation for Question 6.

All included clinical cases corresponded to a single target condition (reversible pulpitis); therefore, no analyses of disease severity or alternative diagnoses were applicable. No clinically relevant time interval or clinical interventions occurred between the index test and the reference standard assessments. As the study was based on predefined clinical cases and repeated model evaluations, a traditional participant flow diagram was not applicable; accordingly, a STARD flow diagram describing clinical case selection and analysis is provided (Figure 1) (Cohen et al., 2016). Baseline demographic characteristics were not applicable, as the unit of analysis was expert-validated clinical cases rather than patients, and no adverse events were associated with the index test or the reference standard.

## 4 Discussion

Until the development of ChatGPT-4, large language models (LLMs) were primarily designed for text-based processing (Meskó, 2023). Consequently, most studies in dentistry have evaluated their performance exclusively on text-derived questions (Mohamad et al., 2022; Balel et al., 2025; Abdulrab et al., 2025; Alsayed et al., 2024; Alan and Alan, 2023). However, the recent incorporation of multimodal capabilities enabling the simultaneous processing of text and images (Elek et al., 2024), has expanded the potential applications of these models. This highlights the need to investigate their performance in image-based diagnostic tasks, as well as to understand their limitations.

The results of this study showed varying levels of accuracy depending on the question. For questions relating to diagnosis that did not require a description of the provided radiograph (Q1-Q4), the accuracy was over 96%. However, for the question that requested a description of the radiographic findings to support the diagnosis (Q6), the accuracy rate was 4.67%. These results suggest that ChatGPT can demonstrate appropriate diagnostic reasoning when given sufficient textual information, but its ability to describe radiographic features is limited. For Question 5, concerning the use of radiographs to establish the diagnosis, the model reported their use in most cases. However, due to the inherent nature of language models, it cannot be determined with certainty whether radiographic information was integrated into the diagnostic process, highlighting the need for further studies assessing diagnostic performance based separately on textual and radiographic data.

When analyzing ChatGPT’s answers to Q6 by differentiating the coronal component (Q6A) and the periapical/pulpal component (Q6B), accuracies of 3.89% and 96.56% were observed. This discrepancy may be due to the complexity of radiographic images. Reversible pulpitis typically lacks periapical/pulpal alterations, whereas coronal findings, particularly carious lesions, are more subtle and therefore more challenging for the model to identify. Regarding the periapical/pulpal descriptions, most answers indicated an intact lamina dura and normal periodontal ligament (PDL) with no radiographic signs of pulpal or periodontal pathology. For example, repetition 2 of Case 1 states: ‘Intact lamina dura, normal and uniform PDL space, no periapical radiolucency, preserved crestal bone, no root resorption or other radiographic signs of apical disease’. In contrast, coronal descriptions frequently failed to identify caries, misclassified their extent or misallocated them on the tooth. Moreover, those graded as partially correct typically lacked a complete description of the coronal structures or used vague terminology (e.g., “proximal”) instead of specifying the exact tooth surface. These results suggest that although ChatGPT shows high accuracy in text-based diagnostic reasoning related to reversible pulpitis, it currently has limited performance in radiographic description tasks for unsupervised clinical use.

In comparison to previous studies that have evaluated ChatGPT’s performance in dental radiograph image analysis, one study (Suárez et al., 2025) also reported limited accuracy. In that study, ChatGPT-4 achieved a score of 38.44% in the interpretation of orthopantomograms for the assessment of lower third molars, simulating clinical requests from patients. In contrast, another study (Durmazpinar and Ekmekci, 2025) evaluated ChatGPT-4 based on case histories, clinical images and radiographs, comparing its answers with those of dental students. ChatGPT achieved an accuracy of 91.4%, outperforming third-year (60.8%) and fifth-year students (79.5%). These contrasting findings highlight the substantial variability in model performance depending on the type of task, the imaging modality, and the evaluation framework. They also underscore the need for further studies to evaluate ChatGPT’s diagnostic accuracy in interpreting dental radiographs.

In terms of repeatability, all questions demonstrated almost perfect repeatability except Q6A, which ranged from substantial to moderate. This reduction in repeatability likely reflects increased variability in the model’s attempts to describe coronal radiographic features, potentially causing the model to produce inconsistent answers. A previous study that used ChatGPT-4 (Suárez et al., 2025) to evaluate third molars on panoramic radiographs also reported moderate repeatability. Together, these results suggest that image-based descriptive tasks may be particularly prone to variability in LLM outputs. Further studies analyzing repeatability of radiographic image interpretation are needed to assess ChatGPT’s performance in image analysis. It is important to highlight that repeatability is an essential metric in evaluating AI performance. For example, Case 6 of the present study illustrates this issue, as the model correctly identified the pathological tooth in only 7 out of 30 repetitions and misidentified it in the remaining 23. Similarly, cases 7 and 10 exhibited a pattern of high but imperfect consistency, with 29 correct identifications and a single incorrect one. Such inconsistencies are commonly known as hallucinations. ‘Hallucinations’ are outputs that sound plausible but are factually incorrect (Alkaissi and McFarlane, 2023). These can create a misleading perception of accuracy (Lee et al., 2023) and pose potential risks in clinical contexts where LLMs are increasingly being considered as decision support tools (Ding et al., 2025; Tangsrivimol et al., 2025). Although newer models, including ChatGPT-5 Thinking, have been promoted as reducing hallucination rates, there is still limited independent empirical evidence (Singh et al., 2026). Our findings emphasize the importance of quantifying repeatability when assessing model performance. Future studies should routinely incorporate repeatability analyses to improve our understanding of the accuracy of LLM-generated outputs.

Comparisons between studies should be interpreted with caution, since performance can be significantly affected by differences in methodology. One relevant factor is the language in which the questions are posed. For example, a recent study found that translating oral pathology multiple-choice questions (MCQs) into English improved ChatGPT-4’s performance compared to the original language version (Wu et al., 2025a). However, a review of different medical exam formats found that translation improved the accuracy of ChatGPT-3.5, but not ChatGPT-4 (Liu et al., 2024). In the present study, the questions were written in English, which is considered the predominant scientific language (Amano et al., 2016; Drubin and Kellogg, 2012). Future studies should examine multilingual performance, given that clinical interactions often take place in the native languages of practitioners.

Another factor affecting performance is the model version. ChatGPT-5 Thinking, which was used in this study, has been presented as a substantial improvement over previous models for use in healthcare applications (Singh et al., 2026). However, scientific evidence supporting this claim remains limited due to its recent release. Previous studies have shown that ChatGPT-4 outperforms ChatGPT-3.5 when answering clinically relevant dental questions (Giannakopoul et al., 2023), orthodontics questions (Makrygiannakis et al., 2024), and U.S. dental examination questions (Dashti et al., 2024). A broader review confirms ChatGPT-4’s superior performance compared with earlier models across healthcare domains (Jin et al., 2024). Similar findings have been reported when comparing ChatGPT-4 and ChatGPT-4o: ChatGPT-4o has been shown to perform better in endodontic MCQs (Arılı Öztürk et al., 2025), in answering clinically relevant endodontic questions (Büker et al., 2025), and in the Chinese National Medical Licensing Examination (Luo et al., 2025). These findings reinforce the need for further studies evaluating newer versions, particularly those designed with enhanced multimodal or healthcare-oriented capabilities.

Prompt design may also influence performance. While prompt engineering has been shown to improve the diagnostic accuracy of ChatGPT-4 in dentistry and medicine (Freire et al., 2024; Wu et al., 2025b), such benefits were not observed in more recent models, including ChatGPT-4.0, ChatGPT-4.1-mini and ChatGPT-4.1 (Hsieh et al., 2025). In this study, a directed, structured prompt was used to guide text- and image-based interpretation. Although this approach enhances methodological consistency, it may limit generalizability to real-world scenarios involving more heterogeneous queries. Future work should therefore assess performance under a wider range of prompt conditions.

Overall, the evidence suggests that variability in LLM performance across studies is due to a combination of factors, including model architecture, prompt design, language and type of task. This highlights the importance of standardizing methodology when evaluating LLMs in clinical and diagnostic contexts.

This study has several limitations that should be considered when interpreting the results. One limitation is related to the diagnostic classification used. The AAE Consensus Conference Recommended Diagnostic Terminology (AAE, 2009) was employed because it is the most widely used system and is likely to be a major source of information for LLM training. However, a new diagnostic classification was introduced in September 2025 (Am Assoc Endodontists, 2026) by the American Association of Endodontists (AAE) and the European Society of Endodontology (ESE). Future studies should therefore assess LLM performance using updated frameworks to ensure continued clinical relevance. Another possible limitation when interpreting the results is that only a single diagnostic condition was evaluated. Therefore, the findings may not be generalizable to other endodontic diagnoses, particularly pulpal conditions such as irreversible pulpitis and pulp necrosis, which may present different clinical and radiographic features. Future studies should evaluate LLM performance across a broader spectrum of endodontic diagnoses.

## 5 Conclusion

When provided with structured clinical information, ChatGPT demonstrated high accuracy in text-based diagnostic reasoning related to reversible pulpitis. However, its performance in radiographic description tasks, particularly those involving coronal structures, was inconsistent. These findings suggest that multimodal AI systems may have the potential to support diagnostic workflows by integrating clinical and radiographic information. However, the current application of ChatGPT in diagnostic workflows should be limited to a supervised decision-support role, particularly when radiographic interpretation is required, to minimize the risk of misinterpretation and ensure patient safety. Clinical judgment, professional responsibility, and appropriate human oversight remain essential for the safe implementation of these technologies in clinical practice.

## Data Availability

The datasets presented in this study can be found in online repositories. The names of the repository/repositories and accession number(s) can be found below: https://osf.io/n4edy/overview.

## References

[B1] AAE. (2009). AAE consensus conference recommended diagnostic terminology. J. Endod. 35 (12), 1634. 10.1016/j.joen.2009.09.035 19932336

[B2] Abdulrab S. Abada H. Mashyakhy M. Mostafa N. Alhadainy H. Halboub E. (2025). Performance of 4 artificial intelligence chatbots in answering endodontic questions. J. Endod. 51 (5), 602–608. 10.1016/j.joen.2025.01.002 39814135

[B3] Alan R. Alan B. M. (2023). Utilizing ChatGPT-4 for providing information on periodontal disease to patients: a DISCERN quality analysis. Cureus 15 (9). 10.7759/cureus.46213 37908933 PMC10613831

[B4] Alayrac J. B. Donahue J. Luc P. Miech A. Barr I. Hasson Y. (2022). Flamingo: a visual language model for few-shot learning. arXiv. 10.48550/arXiv.2204.14198

[B5] Alkaissi H. McFarlane S. I. (2023). Artificial hallucinations in ChatGPT: implications in scientific writing. Cureus 15 (2). 10.7759/cureus.35179 PMC9939079 36811129

[B6] Allihaibi M. Koller G. Mannocci F. (2025). The detection of apical radiolucencies in periapical radiographs: a comparison between an artificial intelligence platform and expert endodontists with CBCT serving as the diagnostic benchmark. Int. Endod. J. 58 (8), 1146–1157. 10.1111/iej.14250 40317970 PMC12254525

[B7] Alsayed A. A. Aldajani M. B. Aljohani M. H. Alamri H. Alwadi M. A. Alshammari B. Z. (2024). Assessing the quality of AI information from ChatGPT regarding oral surgery, preventive dentistry, and oral cancer: an exploration study. Saudi Dent. J. 36 (11), 1483–1489. 10.1016/j.sdentj.2024.09.009 39619711 PMC11605724

[B8] Am Assoc Endodontists (2026). Updating diagnostic terminology in endodontics. Available online at: https://www.aae.org/specialty/diagnostic-terminology-stakeholder-input/ (Accessed June 14, 2026).

[B9] Amano T. González-Varo J. P. Sutherland W. J. (2016). Languages are still a major barrier to global science. PLOS Biol. 14 (12), e2000933. 10.1371/journal.pbio.2000933 28033326 PMC5199034

[B10] Arılı Öztürk E. Turan Gökduman C. Çanakçi B. C. (2025). Evaluation of the performance of ChatGPT‐4 and ChatGPT‐4o as a learning tool in endodontics. Int. Endod. J. 59 (6), 1057–1069. 10.1111/iej.14217 40025853 PMC13158531

[B11] Arruzza E. S. Evangelista C. M. Chau M. (2024). The performance of ChatGPT-4.0o in medical imaging evaluation: a cross-sectional study. J. Educ. Eval. Health Prof. 21, 29. 10.3352/jeehp.2024.21.29 39477547 PMC11586623

[B12] Azad R. Aghdam E. K. Rauland A. Jia Y. Avval A. H. Bozorgpour A. (2022). Medical image segmentation review: the success of U-Net arXiv. Available online at: http://arxiv.org/abs/2211.14830 (Accessed June 14, 2026). 10.1109/TPAMI.2024.3435571 39167505

[B13] Azim A. A. Merdad K. Peters O. A. (2022). Diagnosis consensus among endodontic specialists and general practitioners: an international survey and a proposed modification to the current diagnostic terminology. Int. Endod. J. 55 (11), 1202–1211. 10.1111/iej.13816 35984730 PMC9826047

[B14] Balel Y. Sağtaş K. Teke F. Kurt M. A. (2025). Artificial intelligence‐based detection and numbering of dental implants on panoramic radiographs. Clin. Implant Dent. Relat. Res. 27 (1), e70000. 10.1111/cid.70000 39846131 PMC11755223

[B15] Bjørndal L. Carlsen O. Thuesen G. Darvann T. Kreiborg S. (1999). External and internal macromorphology in 3D‐reconstructed maxillary molars using computerized x‐ray microtomography. Int. Endod. J. 32 (1), 3–9. 10.1046/j.1365-2591.1999.00172.x 10356463

[B16] Büker M. Sümbüllü M. Arslan H. (2025). Comparative performance of chatbots in endodontic clinical decision support: a 4-Day accuracy and consistency study. Int. Dent. J. 75 (5), 100920. 10.1016/j.identj.2025.100920 40720933 PMC12319550

[B17] Cohen J. F. Korevaar D. A. Altman D. G. Bruns D. E. Gatsonis C. A. Hooft L. (2016). STARD 2015 guidelines for reporting diagnostic accuracy studies: explanation and elaboration. BMJ Open 6 (11), e012799. 10.1136/bmjopen-2016-012799 28137831 PMC5128957

[B18] Cotti E. Schirru E. (2022). Present status and future directions: imaging techniques for the detection of periapical lesions. Int. Endod. J. 55 (S4), 1085–1099. 10.1111/iej.13828 36059089

[B19] Dashti M. Ghasemi S. Ghadimi N. Hefzi D. Karimian A. Zare N. (2024). Performance of ChatGPT 3.5 and 4 on U.S. dental examinations: the INBDE, ADAT, and DAT. Imaging Sci. Dent. 54 (3), 271. 10.5624/isd.20240037 39371301 PMC11450412

[B20] Ding L. Fan L. Shen M. Wang Y. Sheng K. Zou Z. (2025). Evaluating ChatGPT’s diagnostic potential for pathology images. Front. Med. 11, 1507203. 10.3389/fmed.2024.1507203 PMC11798939 39917264

[B21] Donnermeyer D. Dammaschke T. Lipski M. Schäfer E. (2023). Effectiveness of diagnosing pulpitis: a systematic review. Int. Endod. J. 56 (S3), 296–325. 10.1111/iej.13762 35536159

[B22] Drubin D. G. Kellogg D. R. (2012). English as the universal language of science: opportunities and challenges. Mol. Biol. Cell 23 (8), 1399. 10.1091/mbc.e12-02-0108 22499829 PMC3341706

[B23] Durmazpinar P. M. Ekmekci E. (2025). Comparing diagnostic skills in endodontic cases: dental students *versus* ChatGPT-4o. BMC Oral Health 25 (1), 457. 10.1186/s12903-025-05857-y 40158110 PMC11955126

[B24] Elek A. Ekizalioğlu D. D. Güler E. (2024). Evaluating microsoft bing with ChatGPT-4 for the assessment of abdominal computed tomography and magnetic resonance images. Diagn Interv. Radiol. 31 (3), 196. 10.4274/dir.2024.232680 39155793 PMC12057540

[B25] Freire Y. Santamaría Laorden A. Orejas Pérez J. Gómez Sánchez M. Díaz-Flores García V. Suárez A. (2024). ChatGPT performance in prosthodontics: assessment of accuracy and repeatability in answer generation. J. Prosthet. Dent. 131 (4), 659.e1–659.e6. 10.1016/j.prosdent.2024.01.018 38310063

[B26] Giannakopoulos K. Kavadella A. Aaqel Salim A. Stamatopoulos V. Kaklamanos E. G. (2023). Evaluation of the performance of generative AI large language models ChatGPT, google bard, and microsoft bing chat in supporting evidence-based dentistry: comparative mixed methods study. J. Med. Internet Res. 25, e51580. 10.2196/51580 38009003 PMC10784979

[B27] Gwet K. L. (2014). “Handbook of inter-rater reliability: the definitive guide to measuring the extent of agreement among raters,” in Advances Analytics. Fourth edition. Gaithersburg, Md: LLC, 410.

[B28] Hsieh M. Y. Wang T. L. Su P. H. Chou M. C. (2025). Impact of prompt engineering on the performance of ChatGPT variants across different question types in medical student examinations: cross-sectional study. JMIR Med. Educ. 11, e78320. 10.2196/78320 41032724 PMC12488032

[B29] Hu M. Qian J. Pan S. Li Y. Qiu R. L. J. Yang X. (2024). Advancing medical imaging with language models: featuring a spotlight on ChatGPT. Phys. Med. Biol. 69 (10), 10TR01. 10.1088/1361-6560/ad387d PMC11075180 38537293

[B30] Innes NPT Frencken J. E. Bjørndal L. Maltz M. Manton D. J. Ricketts D. (2016). Managing carious lesions: consensus recommendations on terminology. Adv. Dent. Res. 28 (2), 49–57. 10.1177/0022034516639276 27099357

[B31] Ismail A. I. Pitts N. B. Tellez M. Authors of the International Caries Classification and Management System (ICCMS) (2015). The international caries classification and management system (ICCMS^TM^) an example of a caries management pathway. BMC Oral Health 15 (S1), S9. 10.1186/1472-6831-15-S1-S9 26391116 PMC4580843

[B32] Issa J. Jaber M. Rifai I. Mozdziak P. Kempisty B. Dyszkiewicz-Konwińska M. (2023). Diagnostic test accuracy of artificial intelligence in detecting periapical periodontitis on two-dimensional radiographs: a retrospective study and literature review. Med. Mex. 59 (4), 768. 10.3390/medicina59040768 PMC10142688 37109726

[B33] Jin H. K. Lee H. E. Kim E. (2024). Performance of ChatGPT-3.5 and GPT-4 in national licensing examinations for medicine, pharmacy, dentistry, and nursing: a systematic review and meta-analysis. BMC Med. Educ. 24 (1), 1013. 10.1186/s12909-024-05944-8 39285377 PMC11406751

[B34] Karkehabadi H. Khoshbin E. Ghasemi N. Mahavi A. Mohammad-Rahimi H. Sadr S. (2024). Deep learning for determining the difficulty of endodontic treatment: a pilot study. BMC Oral Health 24 (1), 574. 10.1186/s12903-024-04235-4 38760686 PMC11102254

[B35] Ker J. Wang L. Rao J. Lim T. (2018). Deep learning applications in medical image analysis. IEEE Access 6, 9375–9389. 10.1109/ACCESS.2017.2788044

[B36] Kung T. H. Cheatham M. Medenilla A. Sillos C. De Leon L. Elepaño C. (2023). Performance of ChatGPT on USMLE: potential for AI-assisted medical education using large language models. PLOS Digit. Health 2 (2), e0000198. 10.1371/journal.pdig.0000198 36812645 PMC9931230

[B37] Lee P. Bubeck S. Petro J. (2023). Benefits, limits, and risks of GPT-4 as an AI chatbot for medicine. N. Engl. J. Med. 388, 1233–1239. 10.1056/NEJMsr2214184 36988602

[B38] Lin X. Hong D. Zhang D. Huang M. Yu H. (2022). Detecting proximal caries on periapical radiographs using convolutional neural networks with different training strategies on small datasets. Diagnostics 12 (5), 1047. 10.3390/diagnostics12051047 35626203 PMC9139265

[B39] Liu M. Okuhara T. Chang X. Shirabe R. Nishiie Y. Okada H. (2024). Performance of ChatGPT across different versions in medical licensing examinations worldwide: systematic review and meta-analysis. J. Med. Internet Res. 26, e60807. 10.2196/60807 39052324 PMC11310649

[B40] Liu Y. Zhang X. Cao W. Cui W. Tan T. Peng Y. (2025). Bootstrapping BI-RADS classification using large language models and transformers in breast magnetic resonance imaging reports. Vis. Comput. Ind. Biomed. Art. 8 (1), 8. 10.1186/s42492-025-00189-8 40178668 PMC11968601

[B41] Lo Giudice R. Nicita F. Puleio F. Alibrandi A. Cervino G. Lizio A. S. (2018). Accuracy of periapical radiography and CBCT in endodontic evaluation. Int. J. Dent. 2018, 1–7. 10.1155/2018/2514243 30410540 PMC6206562

[B42] Luo D. Liu M. Yu R. Liu Y. Jiang W. Fan Q. (2025). Evaluating the performance of GPT-3.5, GPT-4, and GPT-4o in the Chinese national medical licensing examination. Sci. Rep. 15 (1), 14119. 10.1038/s41598-025-98949-2 40269046 PMC12018924

[B43] Makrygiannakis M. A. Giannakopoulos K. Kaklamanos E. G. (2024). Evidence-based potential of generative artificial intelligence large language models in orthodontics: a comparative study of ChatGPT, Google Bard, and Microsoft Bing. Eur. J. Orthod. 13, cjae017. 10.1093/ejo/cjae017 PMC12810200 38613510

[B44] Meskó B. (2023). The impact of multimodal large language models on health care’s future. J. Med. Internet Res. 25, e52865. 10.2196/52865 37917126 PMC10654899

[B45] Mohamad S. F. N. Sukumaran P. Ung N. M. Liew Y. M. (2022). Assessment of demineralized tooth lesions using optical coherence tomography and other state-of-the-art technologies: a review. Biomed. Eng. OnLine 21 (1), 83. 10.1186/s12938-022-01055-x 36463182 PMC9719651

[B46] Najjar R. (2023). Redefining radiology: a review of artificial intelligence integration in medical imaging. Diagnostics 13 (17), 2760. 10.3390/diagnostics13172760 37685300 PMC10487271

[B47] Nirthika R. Manivannan S. Ramanan A. Wang R. (2022). Pooling in convolutional neural networks for medical image analysis: a survey and an empirical study. Neural Comput. Appl. 34 (7), 5321–5347. 10.1007/s00521-022-06953-8 35125669 PMC8804673

[B48] Patel S. Brown J. Semper M. Abella F. Mannocci F. (2019). European society of endodontology position statement: use of cone beam computed tomography in Endodontics: European society of endodontology (ESE) developed by. Int. Endod. J. 52 (12), 1675–1678. 10.1111/iej.13187 31301231

[B49] Raoof M. Vazavandi E. Parizi M. Hatami N. Mohammadalizadeh S. Amanpour S. (2022). Clinical, radiological, and histological correlation in diagnosis of pulpitis. Dent. Res. J. 19 (1), 25. 10.4103/1735-3327.340110 PMC9006151 35432790

[B50] Sabeti M. A. Nikghalb K. Pakzad R. Fouad A. F. (2026). The relationship between pulpal diagnostic conditions and potential inflammatory biomarkers. Int. Endod. J. 59 (1), 23–37. 10.1111/iej.70030 40923157 PMC12701746

[B51] Sau S. George D. D. Singh R. Kohli G. S. Li A. Jalal M. I. (2025). Accuracy and quality of ChatGPT-4o and google gemini performance on image-based neurosurgery board questions. Neurosurg. Rev. 48 (1), 320. 10.1007/s10143-025-03472-7 40131528

[B52] Setzer F. C. Lee S. M. (2021). Radiology in endodontics. Dent. Clin. North Am. 65 (3), 475–486. 10.1016/j.cden.2021.02.004 34051926

[B53] Shieh A. Tran B. He G. Kumar M. Freed J. A. Majety P. (2024). Assessing ChatGPT 4.0’s test performance and clinical diagnostic accuracy on USMLE STEP 2 CK and clinical case reports. Sci. Rep. 14 (1), 9330. 10.1038/s41598-024-58760-x 38654011 PMC11039662

[B54] Silva T. P. Andrade-Bortoletto M. F. S. Ocampo T. S. C. Alencar-Palha C. Bornstein M. M. Oliveira-Santos C. (2024). Performance of a commercially available generative Pre-trained transformer (GPT) in describing radiolucent lesions in panoramic radiographs and establishing differential diagnoses. Clin. Oral Investig. 28 (3), 204. 10.1007/s00784-024-05587-5 38459362 PMC10924032

[B55] Singh A. Fry A. Perelman A. Tart A. Ganesh A. El-Kishky A. (2026). OpenAI GPT-5 system card. arXiv. Available online at: http://arxiv.org/abs/2601.03267 (Accessed June 14, 2026).

[B56] Stephan D. Bertsch A. Burwinkel M. Vinayahalingam S. Al-Nawas B. Kämmerer P. W. (2024). AI in dental radiology—improving the efficiency of reporting with ChatGPT: comparative study. J. Med. Internet Res. 26, e60684. 10.2196/60684 39714078 PMC11704643

[B57] Suárez A. Arena S. Herranz Calzada A. Castillo Varón A. I. Diaz-Flores García V. Freire Y. (2025). Decoding wisdom: evaluating ChatGPT’s accuracy and reproducibility in analyzing orthopantomographic images for third molar assessment. Comput. Struct. Biotechnol. J. 28, 141–147. 10.1016/j.csbj.2025.04.010 40271108 PMC12017887

[B58] Szabó V. Szabó B. T. Orhan K. Veres D. S. Manulis D. Ezhov M. (2024). Validation of artificial intelligence application for dental caries diagnosis on intraoral bitewing and periapical radiographs. J. Dent. 147, 105105. 10.1016/j.jdent.2024.105105 38821394

[B59] Tangsrivimol J. A. Darzidehkalani E. Virk H. U. H. Wang Z. Egger J. Wang M. (2025). Benefits, limits, and risks of ChatGPT in medicine. Front. Artif. Intell. 8, 1518049. 10.3389/frai.2025.1518049 39949509 PMC11821943

[B60] Wu Y. H. Tso K. Y. Chiang C. P. (2025a). Impact of language and question types on ChatGPT-4o’s performance in answering oral pathology questions from Taiwan national dental licensing examinations. J. Dent. Sci. 20 (4), 2176–2180. 10.1016/j.jds.2025.07.010 41040607 PMC12485418

[B61] Wu Y. H. Tso K. Y. Chiang C. P. (2025b). Performance of ChatGPT in answering the oral pathology questions of various types or subjects from Taiwan national dental licensing examinations. J. Dent. Sci. 20 (3), 1709–1715. 10.1016/j.jds.2025.03.030 40654484 PMC12254725

[B62] Zero D. T. Zandona A. F. Vail M. M. Spolnik K. J. (2011). Dental caries and pulpal disease. Dent. Clin. North Am. 55 (1), 29–46. 10.1016/j.cden.2010.08.010 21094717

